# Timely health care seeking and first source of care for acute febrile illness in children in Hawassa, southern Ethiopia

**DOI:** 10.1371/journal.pone.0269725

**Published:** 2022-06-09

**Authors:** Techalew Shimelis, Gill Schierhout, Birkneh Tilahun Tadesse, Sabine Dittrich, John A. Crump, John M. Kaldor, Susana Vaz Nery

**Affiliations:** 1 Kirby Institute, University of New South Wales, Sydney, Australia; 2 College of Medicine and Health Sciences, Hawassa University, Hawassa, Ethiopia; 3 The George Institute for Global Health, University of New South Wales, Sydney, Australia; 4 Foundation for Innovative New Diagnostics, Geneva, Switzerland; 5 Nuffield Department of Medicine, University of Oxford, Oxford, United Kingdom; 6 Centre for International Health, University of Otago, Dunedin, New Zealand; University of Cape Town, SOUTH AFRICA

## Abstract

**Background:**

Timely health care seeking with access to quality health care are crucial to improve child survival. We conducted a study which aimed to identify factors influencing timely health care seeking and choice of first source of health care in Ethiopia.

**Methods:**

A total of 535 caregivers who sought health care for febrile children aged under 5 years at a tertiary hospital, and one urban and two rural health centres in Hawassa, southern Ethiopia were recruited to participate in the study from August to November 2019. Caregivers were interviewed using pretested structured questionnaires on socio-demographic and clinical factors to identify associations with health care seeking practice and first source of care, and reasons for particular practices. Delayed care seeking was defined as seeking care from a health facility after 24 hours of onset of fever.

**Results:**

Of 535 caregivers who participated, 271 (50.7%) had sought timely health care; 400 (74.8%) utilized a primary health care (PHC) facility as first source; and 282 (52.7%) bypassed the nearest PHC facility. Rural residents (adjusted odds ratio (AOR) 1.85; 95% CI 1.11–3.09), and those who reported cough (AOR 1.87; 95% CI 1.20–2.93) as a reason for consultation were more likely to delay seeking health care. While caregivers were less likely delayed for children aged 24–35 months (AOR 0.50; 95% CI 0.28–0.87) compared to infants. Utilizing higher-level hospitals as the first source of care was less frequent among rural residents (AOR 0.15; 95% CI 0.06–0.39) and in those with no formal education (AOR 0.03; 95% CI 0.01–0.27). Those having a longer travel time to the provider (AOR 2.11; 95% CI 1.09–4.08) more likely utilized higher hospitals.

**Conclusion:**

We identified a need to improve timely health seeking among rural residents, infants, and those presenting with respiratory symptoms. Improvements may be achieved by educating communities on the need of early care seeking, and ensuring the communities members’ expectations of services at each level consistent with the services capacity.

## Introduction

Globally, it has been estimated that 5.2 million children aged under 5 years died in 2019, with more than 80% of deaths occurring in sub-Saharan Africa and Central and Southern Asia [1]. Tackling the leading causes of child mortality, specifically pneumonia, diarrhoea, and malaria, all of which commonly present with fever, have been emphasized as a key strategy [1–3]. In Ethiopia, improving health service coverage, with the expansion of health infrastructure and a national health extension program, has contributed to a reduction in mortality of children under-5 years old from 205 to 55 deaths per 1,000 live births between 1990 and 2019 [4]. Despite these gains, Ethiopia has still one of the highest rates of child death and aims to reduce under-5 year old mortality to less than 20 per 1,000 live births by 2035 [5].

To reach the under-5 year old mortality objective, enhancing timely health care seeking behaviour to facilitate early treatment is a crucial component of curative and preventive efforts [6]. Guidelines promote seeking health care within 24 hours of onset of symptoms of febrile illness [2]. For example, Ethiopia’s malaria control and elimination plan aims to achieve a 100% timely health care seeking [7]. However, there is evidence from Ethiopia that only 13.7–38.7% [8–10] seek timely care, and there is inadequate information on the predictors of timely health care seeking practice for childhood febrile illness in African countries [11–14] to plan appropriate interventions.

Moreover, improving access to quality primary health care (PHC) and increasing service utilization enable treatment of illness at an early stage, preventing severe disease and associated hospitalization and death [15]. Proper utilization of PHC facilities also avoids congestion at higher-level hospitals and increases the efficiency of service delivery, reduces cost, and saves time [16]. The success of referral strategy depends on how well PHC is functioning and how informed and adherent are caregivers to the hierarchical referral system [15, 17]. An earlier national report in Ethiopia showed that 74% of febrile children in malarious areas sought care from PHC facilities [18], although little is known about source of care for childhood febrile illness in settings where malaria incidence has declined [19].

In our recent study of consecutive febrile children presenting to a tertiary hospital in Hawassa City, 75.0% had not received any antimicrobial treatment for their illness prior to the hospital visit [20]. Within the same study participants, 38.0% were hospitalized, and 5.9% of those hospitalized died [21]. These data suggested a need to examine health care seeking behaviour in more detail as delay to seeking care from an appropriate source is a key contributor to unfavourable outcomes of childhood febrile illnesses [22]. Caregivers’ decision-making for not utilizing the nearest PHC facilities when the illness is at mild stage, and the practice of bypassing a closer health facilities in preference of distant higher-level health facilities contributes to delays. Therefore, this study aimed to determine proportions of timely care seeking, first source of care utilized, bypassed closer PHC facilities, and reasons for particular practices. We also identified predictors of delays in care seeking and of utilizing higher-level hospitals or PHC facilities as first source of care. Finally, we sought to assess the relationship between timelines of seeking care at first source and hospitalization at tertiary hospital.

## Materials and methods

### Study settings

In Ethiopia, health services are organised into three tiers. Level one is a primary health care unit, including primary hospital, health centre, and health post. Level two is general hospital, and level three is specialized hospital [5]. Health posts are staffed by health extension workers who completed at least the 10^th^ grade and trained for 12 months, and provide promotive, preventive, and curative services including management of malaria, pneumonia, and diarrhoea in rural areas [23]. A two-way referral process ensures a continuum of care between PHC and hospitals and within hospitals and vice-versa [16]. We conducted a prospectively-recruited cross-sectional study during August to November 2019 at health facilities in and around Hawassa City, southern Ethiopia. The city is the capital of Southern Nations and Nationalities Peoples’ Region (SNNPR) and located on Lake Hawassa in the Great Rift Valley. Four health facilities were purposively selected to represent rural and urban settings as well as lower- and higher-level health facilities. They were the largest tertiary public hospital in the administrative region, Hawassa University Comprehensive Specialized Hospital (HUCSH); the nearest urban health centre (HC) to the hospital, Tilte HC; and health centres in two rural areas, Gara-Rikata and Finchawa. While Gara-Rikata is located about 10 km from the Lake, the other three health institutions are located on the banks of the lake.

### Study population

We enrolled caregivers who sought care at the specified health facilities for children aged under 5 years with fever, defined as axillary temperature of at least 37.5°C or a history of fever at least once in the past 48 hours, and of duration no longer than 7 days. Recruitment of participants was undertaken 24 hours a day and 7 days a week. Children who required urgent referral to higher-level care and those whose main reasons for the visit were injury or minor skin infections were excluded. Written consent was obtained from caregivers after provision of information about the study.

### Sample size

The sample size was computed considering timely care seeking (i.e., within 24 hours of illness onset) as the primary outcome. Based on prior Ethiopian data that the proportion of timely care seeking was 38.7% [10], the sample size needed to provide a 95% confidence interval that was of absolute width of 5% was calculated to be 365. In the absence of local information on the prevalence of potential predictors of timely care-seeking, we targeted a sample size such that we had at least 80% power to detect an absolute difference between those who sought timely care and those who did not of 12% or more, across all levels of predictor prevalence. Thus, the overall sample size was determined to be 535. Within this total, we recruited from each health facility in proportion to the recorded attendance numbers of febrile children in the same season in the preceding year. Accordingly, we enrolled 197 participants from the hospital, 138 participants from the urban health centre, and 100 from each of the two rural health centres. Consecutive caregivers of febrile children meeting the inclusion criteria and who consented to participate were enrolled until the allocated sample size was achieved.

### Data collection

#### Caregivers’ interview

Trained study personnel in data collection, who were nurses and health officers, a cadre with 4 years training on clinical and public health services, interviewed consented caregivers to gather data on socio-demographic characteristics of caregivers including sex, age, residence, relationship to the child, occupation, educational status, family size, and the number of children under 5 years old in the household, using structured and pretested questionnaires (S1 Appendix). Data were also collected on age and sex of children. Caregivers were asked the day and time they first recognized the fever. Their response was further refined by asking related questions such as how fever was detected, degree of fever, and child’s general health before onset of fever. Further, duration between onset of fever and seeking care from the first appropriate source of health care, reasons for timely or delayed care seeking, the usual source of health care sought for childhood febrile illness and reasons for the choice, facility utilized as first source for current illness and reasons, means of transport and time to the first health care provider, and if a closer PHC facility based on self-reported proximity was bypassed in seeking care, as well as reasons for bypassing, and child’s symptoms mentioned as a main reason for seeking care from the study facility based on caregivers’ response. Information about particular health seeking practices and reasons was gathered using open-ended questions. Study personnel helped caregivers to recall their care seeking practice by enquiring about related aspects of the event, as appropriate. Data on hospitalization status of children presented to HUCSH and duration of fever were captured.

### Study definitions

Timely care seeking was defined as seeking care from a health facility within 24 hours of onset of fever. Secondary and tertiary hospitals were classified as higher-level hospitals. A caregiver was considered to have bypassed their nearest PHC facility including a health post or health centre or primary hospital if they reported having sought first care from a more distant health facility including higher-level hospitals or another PHC facility or private clinic. The appropriate source of health care was defined as a source authorised to provide evidence-based clinical management service for child illnesses according to the Ethiopian Ministry of Health policy.

### Data analysis

Double data entry and validation were performed using EpiData version-3.1, [24] and analysis done using SPSS version 20 (IBM Corp., Armonk, NY). Proportions were computed to summarize results of categorical variables including socio-demographic characteristics, health care seeking practice, source of care utilized, bypassed PHC, and reasons for various practices. Quantitative data including age, family size, and number of children aged under 5 years in the household were summarized using median and interquartile range (IQR). Crude odds ratios were computed in bivariate logistic regression analysis for initial assessment of the association between binary outcome variables [i.e., seeking first care from a higher-level hospital or other sources (PHC or private clinic), seeking first care from a PHC facility or other sources (higher-hospital or private clinic), delayed or timely health care seeking] and explanatory variables (socio-demographic characteristics and symptoms for seeking care). Adjusted odds ratios (AOR) were calculated via multivariable logistic regression incorporating variables found to have a p-value < 0.20 in bivariate analysis. To check for multicollinearity, we computed the Variance Inflation Factor (VIF) in a regression model using Statistical Analysis System (SAS) version 9.4 (SAS Institute Inc., Cary, NC, USA) where we found no evidence for multicollinearity (VIF ≤ 2). A p-value < 0.05 from multivariable analysis was considered to indicate a significant association.

### Ethical approval

The study was approved by the ethics committees of the University of New South Wales (Ref. No: HC190358) and the Hawassa University College of Medicine and Health Sciences (Ref. No: IRB/223/11).

## Results

### Socio-demographic characteristics of the children and caregivers

A total of 535 caregivers of febrile children were enrolled, 197 (36.8%) were from HUCSH, 138 (25.8%) from Tilte HC, and 100 (18.7%) each from Finchawa HC and Gara-Rikata HC) (S1 Fig). We excluded 9 febrile children who required urgent referral to a higher-level care and one whose main reason for care seeking was skin infection. The median (IQR) age of the children was 18 (9–36) months, and 234 (43.7%) were female. Of 535 interviewed caregivers, 289 (54.0%) were urban residents, 357 (66.7%) were biological mothers, 152 (28.4%) were biological fathers, and 156 (29.2%) had 5–8 grade education (Table 1). The median (IQR) family size and number of under 5 years old children in the household were 5 (4–6) and 1 (1–2), respectively.

**Table 1:** Socio-demographic characteristics of febrile children and caregivers attending health facilities in Hawassa City, 2019 (N = 535).

Characteristics	HUCSH n (%) N = 197	Tilte HC^†^ n (%) N = 138	Finchawa HC^‡^ n (%) N = 100	Gara-Rikata HC^‡^ n (%) N = 100	Total n (%) (N = 535)
**Child age (months)**					
≤11	96 (48.7)	33 (23.9)	21 (21.0)	26 (26.0)	176 (32.9)
12–23	51 (25.9)	32 (23.2)	20 (20.0)	20 (20.0)	123 (23.0)
24–35	26 (13.2)	26 (18.8)	19 (19.0)	22 (22.0)	93 (17.4)
36–47	12 (6.1)	31 (22.5)	25 (25.0)	21 (21.0)	89 (16.6)
48–59	12 (6.1)	16 (11.6)	15 (15.0)	11 (11.0)	54 (10.1)
**Child sex**					
Male	108 (54.8)	67 (48.6)	65 (65.0)	61 (61.0)	301 (56.3)
Female	89 (45.2)	71 (51.4)	35 (35.0)	39 (39.0)	234 (43.7)
**Caregivers’ age (years)**					
≤ 24	62 (31.5)	41 (29.7)	18 (18.0)	13 (13.0)	134 (25.0)
25–34	106 (53.8)	77 (55.8)	50 (50.0)	83 (83.0)	316 (59.1)
≥35	29 (14.7)	20 (14.5)	32 (32.0)	4 (4.0)	85 (15.9)
**Residence**					
Rural	46 (23.4)	0 (0.0)	100 (100)	100 (100)	246 (46.0)
Urban	151 (76.6)	138 (100)	0	0	289 (54.0)
**Caregivers’ relationship to child**					
Biological mother	132 (67.0)	93 (67.4)	49 (49.0)	83 (83.0)	357 (66.7)
Biological father	48 (24.4)	41 (29.7)	48 (48.0)	15 (15.0)	152 (28.4)
Other^1^	17 (8.6)	4 (2.9)	3 (3.0)	2 (2.0)	26 (4.9)
**Caregivers’ occupation**					
Civil servant	62 (31.5)	33 (23.9)	7 (7.0)	5 (5.0)	107 (20.0)
Housewife	11 (5.6)	44 (31.9)	46 (46.0)	79 (79.0)	180 (33.6)
Merchant	23 (11.7)	11 (8.0)	4 (4.0)	1 (1.0)	39 (7.3)
Farmer	41 (20.8)	4 (2.9)	28 (28.0)	10 (10.0)	83 (15.5)
Student	7 (3.6)	15 (10.9)	2 (2.0)	1 (1.0)	25 (4.7)
Self-employed	21 (10.7)	22 (15.9)	13 (13.0)	4 (4.0)	60 (11.2)
Other^2^	32 (16.2)	9 (6.5)	0 (0.0)	0 (0.0)	41 (7.7)
**Caregivers’ educational status**					
No formal schooling	21 (10.7)	11 (8.0)	26 (26.0)	11 (11.0)	69 (12.9)
1–4 grade	22 (11.2)	6 (4.3)	24 (24.0)	38 (38.0)	90 (16.8)
5–8 grade	52 (26.4)	40 (29.0)	34 (34.0)	30 (30.0)	156 (29.2)
9–12 grade	43 (21.8)	45 (32.6)	10 (10.0)	15 (15.0)	113 (21.1)
Higher education	59 (29.9)	36 (26.1)	6 (6.0)	6 (6.0)	107 (20.0)
**N****o** **of under-5 children in household**					
1 child	119 (60.4)	99 (71.7)	51 (51.0)	84 (84.0)	353 (66.0)
2 children	65 (33.0)	37 (26.8)	48 (48.0)	16 (16.0)	166 (31.0)
≥3 children	13 (6.6)	2 (1.4)	1 (1.0)	0 (0.0)	16 (3.0)

^1^Grandfather/mother (n = 5), uncle/aunt (n = 6), brother/sister (n = 7), other relation (n = 3), no relation (n = 5)

^2^No work (n = 20), daily labourer (n = 13), other (n = 8)

^†^ Urban health centre;

^‡^ Rural health centre

### Caregivers’ timelines of seeking health care

Timely care was sought for 271 (50.7%) of 535 children. As shown in Table 2, caregivers of children aged 24–35 months were less likely to delay seeking care (AOR 0.50; 95% CI 0.28–0.87) compared to those aged ≤11 months, and rural residents (AOR 1.85; 95% CI 1.11–3.09) more frequently sought delayed care compared to urban residents. However, caregivers who mentioned cough (AOR 1.87; 95% CI 1.20–2.93) as a reason for consultation more frequently sought delayed care compared to those who did not. While bypassing the nearest PHC was significantly associated with a delayed care in bivariate analysis, it was not significantly associated in multivariable analysis (AOR 1.47; 95% CI 0.94–2.30). The main reasons given by 271 caregivers for timely care seeking were to avoid complications of illness, reported by 218 (80.4%), and severe illness, reported by 110 (40.6%). Of 264 caregivers whose care seeking was delayed, 166 (62.9%) said that the illness was mild, 104 (39.4%) reported being unaware of the need to seek care within 24 hours of onset of fever, and 87 (33%) mentioned lack of money (Table 3).

**Table 2:** Factors influencing the odds of delayed (after 24 hours of onset of fever) in health care seeking for febrile children in Hawassa City, 2019 (N = 535).

Characteristics	Timing of seeking care after onset of fever		COR (95% CI)	P-value	AOR (95% CI)	P-value
	≤ 24 h n (%)^3^	> 24 h n (%)^3^				
**Child age (months)**						
≤11	73 (41.5)	103 (58.5)	1		1	
12–23	68 (55.3)	55 (44.7)	0.57 (0.36–0.91)	0.019	0.63 (0.38–1.05)	0.078
24–35	56 (60.2)	37 (39.8)	0.47 (0.28–0.78)	0.004	**0.50 (0.28–0.87)**	0.015
36–47	48 (53.9)	41 (46.1)	0.61 (0.36–1.01)	0.055	0.72 (0.40–1.29)	0.267
48–59	26 (48.1)	28 (51.9)	0.76 (0.41–1.41)	0.387	0.87 (0.44–1.73)	0.691
**Residence**						
Urban	176 (60.9)	113 (39.1)	1		1	
Rural	95 (38.6)	151 (61.4)	2.48 (1.75–3.51)	<0.001	**1.85 (1.11–3.09)**	0.019
**Caregivers’ Occupation**						
Civil servant	66 (61.7)	41 (38.3)	1		1	
Housewife	86 (47.8)	94 (52.2)	1.76 (1.08–2.86)	0.023	1.16 (0.57–2.37)	0.681
Merchant	19 (48.7)	20 (51.3)	1.69 (0.81–3.55)	0.162	1.41 (0.58–3.45)	0.449
Farmer	25 (30.1)	58 (69.9)	3.74 (2.03–6.87)	<0.001	2.26 (0.98–5.20)	0.059
Student	17 (68.0)	8 (32.0)	0.76 (0.30–1.91)	0.557	0.78 (0.29–2.15)	0.636
Self-employed	39 (65.0)	21 (35.0)	0.87 (0.45–1.67)	0.670	0.72 (0.32–1.60)	0.419
Other^1^	19 (46.3)	22 (53.7)	1.86 (0.90–3.86)	0.093	1.72 (0.72–4.10)	0.219
**Caregivers’ educational status**						
No formal education	28 (40.6)	41 (59.4)	2.36 (1.27–4.38)	0.007	0.98 (0.41–2.36)	0.970
1–4 grade	32 (35.6)	58 (64.4)	2.92 (1.63–5.22)	<0.001	1.53 (0.68–3.43)	0.301
5–8 grade	77 (49.4)	79 (50.6)	1.65 (1.00–2.72)	0.049	0.99 (0.49–1.98)	0.966
9–12 grade	68 (60.2)	45 (39.8)	1.07 (0.62–1.83)	0.819	0.77 (0.39–1.51)	0.439
Higher education	66 (61.7)	41 (38.3)	1		1	
**Travel time to first care**^2^ **(minute)**						
≤15	167 (54.6)	139 (45.4)	1		1	
16–30	84 (48.6)	89 (51.4)	1.27 (0.88–1.85)	0.205	0.99 (0.65–1.51)	0.951
31–45	10 (47.6)	11 (52.4)	1.32 (0.55–3.20)	0.537	0.61 (0.22–1.69)	0.343
≥46	10 (28.6)	25 (71.4)	3.00 (1.40–6.47)	0.005	1.48 (0.62–3.49)	0.375
**Bypassed a nearer PHC**						
No	154 (60.9)	99 (39.1)	1		1	
Yes	117 (41.5)	165 (58.5)	2.19 (1.55–3.10)	<0.001	1.47 (0.94–2.30)	0.092
**Reasons (symptoms) for consultation at current facility** ^¶^						
**Cough**						
No	160 (57.6)	117 (42.2)	1		1	
Yes	111 (43.2)	147 (57.0)	1.81 (1.29–2.55)	0.001	**1.87 (1.20–2.93)**	0.006
**Fast breathing**						
No	234 (52.0)	216 (48.0)	1		1	
Yes	37 (43.5)	48 (56.5)	1.41 (0.88–2.24)	0.153	0.91 (0.48–1.72)	0.767
**Sweating**						
No	261 (52.2)	239 (47.8)	1		1	
Yes	10 (28.6)	25 (71.4)	2.73 (1.28–5.80)	0.009	1.59 (0.65–3.92)	0.312
**Refuse to drink**						
No	259 (51.7)	242 (48.3)	1		1	
Yes	12 (35.3)	22 (64.7)	1.96 (0.95–4.05)	0.068	2.10 (0.93–4.76)	0.075
**N****o** **of symptoms for consultation**						
1	40 (63.5)	23 (36.5)	1		1	
2	122 (51.9)	113 (48.1)	1.61 (0.91–2.86)	0.103	1.10 (0.58–2.10)	0.765
3	80 (51.6)	75 (48.4)	1.63 (0.89–2.98)	0.111	1.10 (0.54–2.25)	0.795
≥4	29 (35.4)	53 (64.6)	3.18 (1.60–6.30)	0.001	1.92 (0.73–5.02)	0.186

m, month; h, hour; PHC, primary health care; COR, crude odds ratio; AOR, adjusted odds ratio; CI, confidence interval

^**¶**^ Multiple response possible;

^1^No work (n = 20), daily labourer (n = 13), other (n = 8),

^2^Travel time to the first accessed source of care by most used transport means;

^3^Percentages within categories of the characteristics (raw total)

**Table 3:** Caregivers’ reasons for timely (within 24 hours of onset of fever) and delayed (after 24 hours of onset of fever) care seeking for child’s fever episode in Hawassa City, 2019.

Caregivers’ reasons^¶^	Frequency (%)
**Timely care seeking (N = 271)**	
To avoid complication	218 (80.4)
Severe illness	110 (40.6)
Aware of timely care seeking	61 (22.5)
Availability of a closer facility	45 (16.6)
Experience	39 (14.4)
Advised by others	25 (9.2)
Other^§^	3 (1.1)
**Delayed care seeking (N = 264)**	
Mild illness	166 (62.9)
Unaware about timely care seeking	104 (39.4)
Shortage of money	87 (33.0)
Busy with other duties	25 (9.5)
Family would not let	15 (5.7)
Poor transport access	17 (6.4)
No closer facility	10 (3.8)

^§^ Fear of malaria (n = 2), refuse to eat or drink (n = 1)

^**¶**^ Multiple response possible

### Source of health care for febrile illness

Of the 535 participating caregivers, 307 (57.4%) reported knowing where to seek first health care for febrile children under 5 years of age. Of 535 participants, 410 (76.6%) reported usually utilizing health centres as first source of care, while 55 (10.3%) reported using higher-level hospitals. The main reasons given were proximity, mentioned by 315 (58.9%) caregivers; and availability of skilled staff by 253 (47.3%), a laboratory service by 229 (42.8%), and drugs by 125 (23.4%).

**Fig 1:**
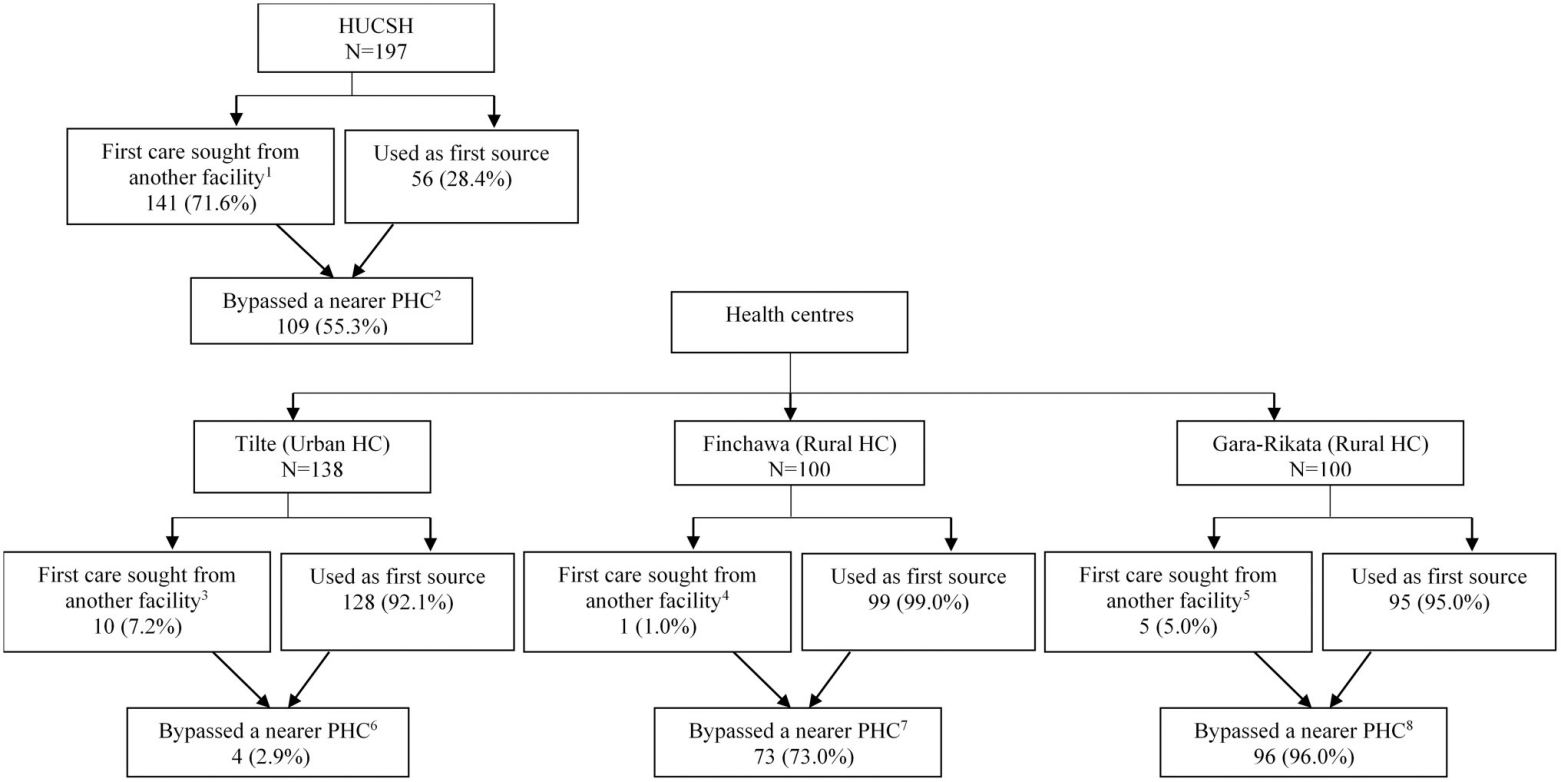
First source of care utilized and bypassed primary health care by caregivers at the study health facilities in Hawassa City, 2019. HUCSH; Hawassa University Comprehensive Specialized Hospital; PHC, primary health care; HC, health centre ^1^ [Secondary hospital (n = 36), primary hospital (n = 11), health centre (n = 55), health post (n = 3), private clinic (n = 36)] ^2^ [Primary hospital (n = 3), health centre (n = 86); health post (n = 20)] ^3^ [Higher-level hospitals (n = 1), health centre (n = 5), private clinic (n = 4)]; ^4^ [health post (n = 1)]; ^5^ [health centre (n = 3), private clinic (n = 2)] ^6^ [Primary hospital (n = 3), health centre (n = 1)]; ^7^ [health post (n = 73)]; ^8^ [health centre (n = 19), health post (n = 77)].

For the current episode, 400 (74.8%) of 535 sought care first from PHC facilities; 385 (72.0%) children from a health centre, 11 (2.1%) from a primary hospital, and 4 (0.7%) from a health post. The reminder (93, 17.4%) caregivers sought first care from higher-level hospitals: 56 (10.5%) from tertiary and 37 (6.9%) from secondary facilities, and 42 (7.9%) from private clinics. As shown in Fig 1, 141 (71.6%) of 197 caregivers presenting at HUCSH sought first care from other facilities: 36 (18.3%) from a secondary hospital, 11 (5.6%) from a primary hospital, 55 (27.9%) from a secondary hospital, 3 (1.5%) from a health post, and 36 (18.3%) from a private clinic. Among caregivers recruited at Tilte, Finchawa, and Gara-Rikata health centres, 128 (92.1%), 99 (99.0%), and 95 (95.0%) sought first care for the episode at these clinics, respectively.

### Bypassing a PHC facility

A total of 282 (52.7%) of 535 caregivers reported to having a PHC facility (HP, HC, or primary hospital) nearer than the one utilized as first source during the current episode of child illness. Of 93 caregivers who sought first care from higher-level hospitals, 64 (68.8%) bypassed a nearer PHC facility, which was a health centre for 55 (85.9%) of 64. Of 64 bypassed facilities, 35 (54.7%) were within 15-minute walk from caregivers’ homes. The main reasons for bypassing were perceived lack of skilled staff, mentioned by 23 (35.9%) caregivers, lack of trust (31.2%), and lack of medical equipment (29.7%). Of 400 caregivers who sought first care from a PHC facility, 191 (47.8%) reported bypassing their nearest PHC facility. Of these bypassed facilities, 163 (85.3%) were health posts and 26 (13.6%) were health centres. The main reasons were perceived lack of laboratory service, 153 (80.1%), drugs, 117 (61.3%), or skilled staff, 114 (59.7%). Of 42 caregivers who sought first care from a private clinic, 27 (64.3%) bypassed a nearer PHC facility. Most (158, 82.7%) bypassed facilities were located within 15-minute walking distance from caregivers’ homes (S1 Table).

### Socio-demographic and clinical factors associated with first source of care

Table 4 shows associations between socio-demographic and clinical factors, and utilization of higher-level hospitals and PHC facilities. In multivariable logistic regression analysis, utilization of higher-level hospitals as first source of care was less common among rural (AOR 0.15; 95% CI 0.06–0.39) compared to urban residents, and among respondents who were the biological fathers of febrile children (AOR 0.42; 95% CI 0.21–0.85) compared to mothers; housewives (AOR 0.09; 95% CI 0.03–0.30) or students (AOR 0.17; 95% CI 0.04–0.74) compared to civil servants; and those with no formal education (AOR 0.03; 95% CI 0.01–0.27) compared to those with higher education level (above secondary school). Respondents who more likely sought first care from higher-level hospitals were those having a travel time 16–30 minutes (AOR 2.11; 95% CI 1.09–4.08) or ≥31 minutes (AOR 11.3; 95% CI 3.99–31.9) to get to the provider by commonly used transport means compared to those with a travel time of ≤15 minutes. Caregivers who mentioned fever as a reason for consultation (AOR 0.14; 95% CI 0.02–0.78) compared those who did not mention fever were less likely to have utilized higher-level hospitals as first source of care, as were those who mentioned only one symptom (AOR 0.11; 95% CI 0.02–0.54) compared to those who mentioned at least 4 symptoms.

**Table 4:** Factors associated with seeking care first at higher-level hospitals and PHC for febrile children in Hawassa City, 2019.

Characteristics	Higher-level hospitals n (%)^2^	COR (95% CI)	P-value	AOR (95% CI)	P-value	PHC facilities n (%)^2^	COR (95% CI)	P-value	AOR (95% CI)	P-value
**Child age (month)**										
≤11	41 (23.3)	1		1		118 (67.0)	1		1	
12–23	23 (18.7)	0.76 (0.43–1.34)	0.341	0.69 (0.33–1.44)	0.325	87 (70.7)	1.19 (0.72–1.96)	0.499	1.50 (0.75–3.01)	0.256
24–35	12 (12.9)	0.49 (0.24–0.98)	0.044	0.85 (0.34–2.09)	0.719	72 (77.4)	1.69 (0.95–3.01)	0.077	0.94 (0.43–2.09)	0.884
36–47	9 (10.1)	0.37 (0.17–0.80)	0.012	0.83 (0.30–2.30)	0.721	78 (87.6)	3.49 (1.72–7.06)	0.001	2.33 (0.87–6.23)	0.093
48–59	8 (14.8)	0.57 (0.25–1.31)	0.187	1.05 (0.36–3.03)	0.929	45 (83.3)	2.46 (1.13–5.37)	0.024	1.39 (0.50–3.88)	0.533
**Residence**										
Urban	80 (27.7)	1		1		172 (59.5)	1		1	
Rural	13 (5.3)	0.15 (0.08–0.27)	<0.001	**0.15 (0.06–0.39)**	<0.001	228 (92.7)	8.62 (5.05–14.7)	<0.001	**6.50 (2.92–14.4)**	<0.001
**Caregivers’ relationship to child**										
Biological mother	59 (16.5)	1		1		268 (75.1)	1		1	
Biological father	21 (13.8)	0.81 (0.47–1.39)	0.442	**0.42 (0.21–0.85)**	0.015	121 (79.6)	1.30 (0.82–2.06)	0.271	**3.09 (1.59–6.02)**	0.001
Other	13 (50.0)	5.05 (2.23–11.4)	<0.001	3.09 (0.91–10.6)	0.072	11 (42.3)	0.24 (0.11–0.55)	0.001	**0.23 (0.06–0.93)**	0.039
**Caregivers’ occupation**										
Civil servant	38 (35.5)	1		1		56 (52.3)	1		1	
Housewife	6 (3.3)	0.06 (0.03–0.16)	<0.001	**0.09 (0.03–0.30)**	<0.001	169 (93.9)	14.0 (6.82–28.7)	<0.001	**5.43 (2.07–14.2)**	0.001
Merchant	7 (17.9)	0.40 (0.16–0.99)	0.046	0.33 (0.10–1.09)	0.068	26 (66.7)	1.82 (0.85–3.92)	0.125	1.20 (0.41–3.35)	0.736
Farmer	16 (19.3)	0.43 (0.22–0.85)	0.015	0.89 (0.28–2.79)	0.841	65 (78.3)	3.29 (1.73–6.27)	<0.001	0.77 (0.26–2.32)	0.644
Student	4 (16.0)	0.35 (0.11–1.08)	0.068	**0.17 (0.04–0.74)**	0.018	20 (80.0)	3.64 (1.27–10.4)	0.016	**7.42 (1.85–29.8)**	0.005
Private work	9 (15.0)	0.32 (0.14–0.72)	0.006	0.44 (0.16–1.22)	0.114	44 (73.3)	2.50 (1.26–4.97)	0.009	1.11 (0.42–2.93)	0.833
Other	13 (31.7)	0.84 (0.39–1.82)	0.663	0.55 (0.19–1.57)	0.261	20 (48.8)	0.87 (0.42–1.78)	0.699	0.70 (0.26–1.92)	0.489
**Caregivers’ educational status**										
No formal education	2 (2.9)	0.08 (0.02–0.33)	0.001	**0.03 (0.01–0.27)**	0.001	64 (92.8)	1		1	
1–4 grade	11 (12.2)	0.36 (0.17–0.76)	0.008	0.93 (0.28–3.14)	0.904	78 (86.7)	0.51 (0.17–1.52)	0.225	**0.11 (0.02–0.49)**	0.004
5–8 grade	26 (16.7)	0.51 (0.28–0.93)	0.028	0.87 (0.35–2.18)	0.769	123 (78.8)	0.29 (0.11–0.78)	0.014	**0.08 (0.02–0.34)**	0.001
9–12 grade	24 (21.2)	0.69 (0.37–1.28)	0.243	0.83 (0.35–1.94)	0.662	78 (69.0)	0.17 (0.06–0.47)	0.001	**0.06 (0.01–0.27)**	<0.001
Higher education	30 (28.0)	1		1		57 (53.3)	0.09 (0.03–0.24)	<0.001	**0.03 (0.01–0.12)**	<0.001
**Travel time to first care1 (minute)**										
≤15	45 (14.7)	1		1		233 (76.1)	1		1	
16–30	29 (16.8)	1.17 (0.70–1.94)	0.550	**2.11 (1.09–4.08)**	0.027	133 (76.9)	1.04 (0.67–1.62)	0.856	0.58 (0.31–1.08)	0.085
≥31	19 (33.9)	2.98 (1.58–5.63)	0.001	**11.3 (3.99–31.9)**	<0.001	34 (60.7)	0.48 (0.27–0.88)	0.017	**0.14 (0.05–0.40)**	<0.001
**No of under-5 children in household**										
1	60 (17.0)	1		1		266 (75.4)	1		1	
2	25 (15.1)	0.87 (0.52–1.44)	0.578	1.09 (0.57–2.11)	0.793	128 (77.1)	1.10 (0.71–1.70)	0.663	0.91 (0.50–1.67)	0.762
≥3	8 (50.0)	4.88 (1.76–13.5)	0.002	3.42 (0.86–13.6)	0.082	6 (37.5)	0.20 (0.07–0.56)	0.002	**0.21 (0.05–0.95)**	0.043
**Reasons (symptoms) for consultation**										
Fever										
No	5 (33.3)	1		1		9 (60.0)	1		1	
Yes	88 (16.9)	0.41 (0.14–1.22)	0.109	**0.14 (0.02–0.78)**	0.025	391 (75.2)	2.02 (0.71–5.79)	0.190	**7.10 (1.48–33.9)**	0.014
Cough										
No	31 (11.2)	1		1		228 (82.3)	1		1	
Yes	62 (24.0)	2.51 (1.57–4.02)	<0.001	1.36 (0.68–2.72)	0.384	172 (66.7)	0.43 (0.29–0.64)	<0.001	1.06 (0.55–2.07)	0.855
Fast breathing										
No	67 (14.9)	1		1		361 (80.2)	1		1	
Yes	26 (30.6)	2.52 (1.48–4.28)	0.001	0.95 (0.42–2.17)	0.911	39 (45.9)	0.21 (0.13–0.34)	<0.001	**0.42 (0.19–0.91)**	0.028
Shivering										
No	92 (19.5)	1		1		338 (71.8)	1		1	
Yes	1 (1.6)	0.07 (0.01–0.48)	0.007	0.12 (0.02–1.02)	0.052	62 (96.9)	12.2 (2.94–50.6)	0.001	**7.56 (1.48–38.5)**	0.015
Sweating										
No	86 (17.2)	1				381 (76.2)	1		1	
Yes	7 (20.0)	1.20 (0.51–2.85)	0.673	N/A	-	19 (54.3)	0.37 (0.19–0.74)	0.005	**0.27 (0.08–0.93)**	0.038
Multiple symptoms										
1	3 (4.8)	0.12 (0.04–0.42)	0.001	**0.11 (0.02–0.54)**	0.007	57 (90.5)	8.62 (3.35–22.2)	<0.001	**6.34 (1.62–24.8)**	0.008
2	30 (12.8)	0.35 (0.19–0.65)	0.001	0.40 (0.15–1.08)	0.071	190 (80.9)	3.83 (2.23–6.58)	<0.001	1.67 (0.62–4.52)	0.311
3	36 (23.2)	0.73 (0.40–1.34)	0.310	0.62 (0.26–1.46)	0.276	110 (71.0)	2.22 (1.27–3.86)	0.005	1.80 (0.76–4.26)	0.179
≥4	24 (29.3)	1		1		43 (52.4)	1		1	

n, number; m, month; gr, grade; PHC, primary health care; AOR, adjusted odds ratio; CI, confidence interval

^1^Travel time to the first accessed source of care by most used transport means;

^2^Percentages within categories of the characteristics (raw total)

N/A, not applicable (a variable with p-value > 0.20 and not included in the multivariable analysis)

On the other hand, caregivers who mentioned child’s shivering (AOR 7.10; 95% CI 1.48–33.9) or sweating (AOR 7.56; 95% CI 1.48–38.5) as a reason for consultation more likely sought first care from PHC facilities, while those that mentioned fast breathing (AOR 0.42; 95% CI 0.19–0.91) less frequently utilized first care from PHC facilities.

The main reasons mentioned by 93 caregivers who used higher-level hospitals as first source of care for current episode of fever were perceived availability of skilled staff for 66 (71.0%), trusted service for 38 (40.9%), and nearest facility for 35 (37.6%). Of 400 caregivers who sought first care from a PHC facility, the main reasons given were nearest facility for 239 (59.8%), availability of laboratory service for 195 (48.8%), and presence of skilled staff for 188 (47.0%) (Table 5).

**Table 5:** Perceived reasons for source of care first utilized for the current episode of illness in Hawassa City, 2019 (N = 493).

Reasons for source of care first utilized^¶^	Higher-level hospitals n (%) (N = 93)	PHC facilities n (%) (N = 400)
Nearest facility	35 (37.6)	239 (59.8)
Availability of laboratory service	23 (24.7)	195 (48.8)
Availability of skilled staff	66 (71.0)	188 (47.0)
Affordable cost	26 (28.0)	143 (35.8)
Shorter waiting time	3 (3.2)	133 (33.2)
Availability of drugs	13 (14.0)	132 (33.0)
Availability of medical equipment	29 (31.2)	78 (19.5)
Trusted service	38 (40.9)	66 (16.5)
Familiar with the provider	27 (29.0)	56 (14.0)
Advised by colleague	6 (6.5)	31 (7.8)
Availability of admission facility	16 (17.2)	0

PHC, primary health care; AOR, adjusted odds ratio; CI, confidence interval

^**¶**^ Multiple response possible

### Relationship between timelines of seeking care at first source and hospitalization

We compared the likelihood of being hospitalised at HUCSH for children who had a timely presentation versus those with delayed presentation at first source (S2 Table). For those with delayed presentation, there was a four-fold increased odds of hospitalisation for those who first sought care at a PHC facility compared to those who first sought care at higher-level hospitals (crude odds ratio [COR] 4.09; 95% CI 1.54–10.8). For those with a timely presentation, there was no significant difference in the odds of hospitalisation for those who sought first care at the PHC facility compared to those at higher-level hospitals (COR 1.23; 95% CI 0.41–3.70).

## Discussion

Our study showed that around half of the participants sought health care within 24 hours of the fever onset, three quarters sought first care from a PHC facility, and over half bypassed the nearest PHC facility in preference for higher-level hospitals or different PHC facilities. Care-seeking was more likely delayed by rural residents, caregivers of infants, and those who mentioned cough as a reason for consultation. Higher-level hospitals were less likely to be utilized as first care by rural residents and those with no formal education.

The proportion of children for whom health care seeking was within 24 hours (50.7%) was higher than results of community-based studies in febrile children aged under 5 years in Ethiopia (13.7–38.7%) [8–10]. The study design might contribute to the observed difference as a comparable finding (51.4%) to ours was reported in a recent hospital-based study in children with pneumonia in northwest Ethiopia [25]. While the proportion of timely care seeking in febrile children in Uganda (48.8%) [14] was similar to our result, lower findings (40.7–44.6%) [11–13] were also reported elsewhere.

Our findings indicate that caregivers of febrile children from rural areas more frequently delay seeking health care, as has also been reported for children with pneumonia in Ethiopia [25]. However, our observation of lower odds of delay in care seeking for children aged 24–35 months was in contrast to a finding from Tanzania where timely care was more frequently sought for infants [12]. This might be due to differences between countries in caregivers’ responses to specific, age-related presenting conditions. We observed that caregivers who mentioned cough as a reason for consultation were more likely to have delayed in seeking care, while other studies from African countries reported delays among children without fast breathing [14] or diarrhoea [12]. These studies also identified longer distance to the care provider [12, 14], wet season [11], lack of awareness about early health care seeking [13, 25], and lower socio-economic status [8, 14, 25] as being associated with delayed care seeking. However, we did not find an association with travel time, which may be viewed as a surrogate for distance to the provider. Moreover, the perception that the illness was not severe, lack of awareness about timely health care seeking, and lack of finance were given as main reasons for the delay in our study, consistent to a report from Malawi [26]. Thus, it is crucial to educate communities about the need of early health care seeking, particularly for respiratory symptoms in infants, which might be perceived as mild illness, but progressing to severe pneumonia.

The majority of caregivers (74.8%) sought first care from a PHC facility, consistent with findings from a recent national survey in Ethiopia that children with fever in malarious areas sought care from health centres (49.5%) or health posts (24.9%) [18]. The proportion was lower than a result (54.0%) from demographic and health survey in Tanzania [27]. The studies also showed 6.3 and 7.4% of febrile children utilized a higher-level hospital as first source of care [18, 27], which were lower than our finding (17.4%). Urban residents and those with a higher level of education more likely sought first care from a higher-level hospital in contrast to a PHC facility that was commonly attended by rural residents and those with lower level of education, as reported elsewhere [27]. This is possibly because wealthier caregivers tended to seek a perceived higher quality care at higher-level hospitals, possibly after having a longer travel time to the provider, as observed in the current study. Multiple or illness-specific symptoms may influence caregivers’ choice for level of care, as observed for shivering and fast breathing that are frequently associated with malaria and pneumonia, respectively. As compared to caregivers who utilized PHC as first source, higher proportions of those who used higher-level hospitals mentioned the availability of skilled staff (47.0% versus 71.0%) and a more trusted service (16.5% versus 40.9%) as reasons for their choice.

We found that around half of the caregivers bypassed the nearest PHC facility, a similar proportion to that reported from Tanzania (59.2%) [15]. Caregivers’ perceptions related to quality of care including unavailability of skilled staff and lack of laboratory service or drugs were mentioned as reasons for bypassing health posts in favour of health centres, and PHC facilities in favour of higher-level hospitals. Similar findings of lack of quality service were reported in health posts in Ethiopia [28] and PHC facility in Tanzania [15]. While bypassing a PHC facility was more frequent with lower travel time to the district hospital in Tanzania [15], our study identified a longer travel time as a predictor for seeking first care from higher-level hospitals. This suggests that closer PHC facilities were more frequently bypassed for a perceived higher quality service at a more distant facility, despite additional time and costs. The observed high proportion of bypassing reflects that caregivers had less access to the health services, when and where they need them, contributing to delay in seeking care. Moreover, the difficulty to access a perceived quality service at distant health facilities for caregivers with low socio-economic status is an equity issue.

A qualitative research to better understand communities’ needs and expectations from PHC facilities help plan measures that improve access to quality care and may reduce bypassing and delays. Further, an assessment of quality of care at relevant facilities would presumably help in case some caregivers’ perceptions are justified. In fact, several challenges to the implementation of integrated community case management strategy have been reported in a recent systematic review in Ethiopia [23], such as lack of essential medications for pneumonia in southern Ethiopia [29]. Bypassing health posts by nearly all caregivers at rural health canters, especially in high malaria risk areas such as Finchawa, is a major concern to be addressed by ensuring the availability of malaria rapid diagnostic tests and antimalarial drugs at health posts to improve caregivers’ trust.

The observed similar proportions of hospitalized children among those who sought timely care at PHC facilities and higher-level hospitals may indicate that early care seeking at PHC facilities can deliver good patient outcomes, as reported in other studies [30, 31]. However, a higher proportion of hospitalization among those who had sought delayed care at PHC facilities may be due to additional delays before reaching a tertiary hospital, as also reflected by median duration of fever on hospitalization (3 versus 5 days). Likewise, besides home delays, accessing higher-level facilities and transport delays were reported as determinants of child mortality [22].

The study faced some limitations in light of which results need to be interpreted. As we recruited in selected health facilities, we cannot compare our participants with those of febrile children who were never brought to a facility. Also, we did not recruit at all health facilities in Hawassa, but made efforts to enhance the generalizability of our findings by including facilities at higher and lower levels of care and in both rural and urban settings. We should acknowledge that our study has only provided information on appropriate sources of care, and leaves open the question of the role of inappropriate sources of care in timeliness of care seeking. Our data collection relied on caregivers’ report, and might be subject to information and recall bias.

## Conclusion

We found that a high proportion of children under 5 years old with febrile illnesses attending health facilities in a mid-sized Ethiopian urban centre were brought to a health facility more than 24 hours after the onset of illness. Most likely to present with delay were rural residents, infants, and those with a cough as the primary reason for consultation. Most caregivers utilized a PHC facility as first source of care although a closer health post was often bypassed. Our findings emphasize the need for educating communities on early care seeking and addressing community expectations in the provision of care, especially at health posts through strengthening the implementation of integrated community case management. A better understanding is also needed of caregivers’ perceptions of their health service, whether their perceptions are rational, and how to build their confidence in existing referral pathways.

## Supporting information

S1 Fig Participant screening and enrolment, conducted at the study health facilities in Hawassa City, 2019. HUCSH, Hawassa University Comprehensive Specialized Hospital; HC; health centre. Reason for exclusion: ^1^ Skin infection, ^2^ Urgent referral to higher-level care.
(TIF)

S1 Table Bypassed nearest PHC and perceived reasons for bypassing during the current fever episode in Hawassa City Administration, 2019 (N = 535). ^⸹^Any nearest primary health care facility different from the one first attended ^1^Travel time to a bypassed primary health care facility by walking.
(DOCX)

S2 Table Relationship between timelines of seeking care at first source and hospitalization at HUCSH, 2019. PHC, primary health care; COR, crude odds ratio; AOR, adjusted odds ratio; CI, confidence interval ^1^Percentages within categories of the characteristics (raw total).
(DOCX)

S1 Appendix Questionnaire. (PDF)
