# Supplementary figures and images for: Timely health care seeking and first source of care for acute febrile illness in children in Hawassa, southern Ethiopia
Source: PLoS One. 2022 Jun 9;17(6):e0269725. doi: 10.1371/journal.pone.0269725 (PMC9182269; doi:10.1371/journal.pone.0269725)

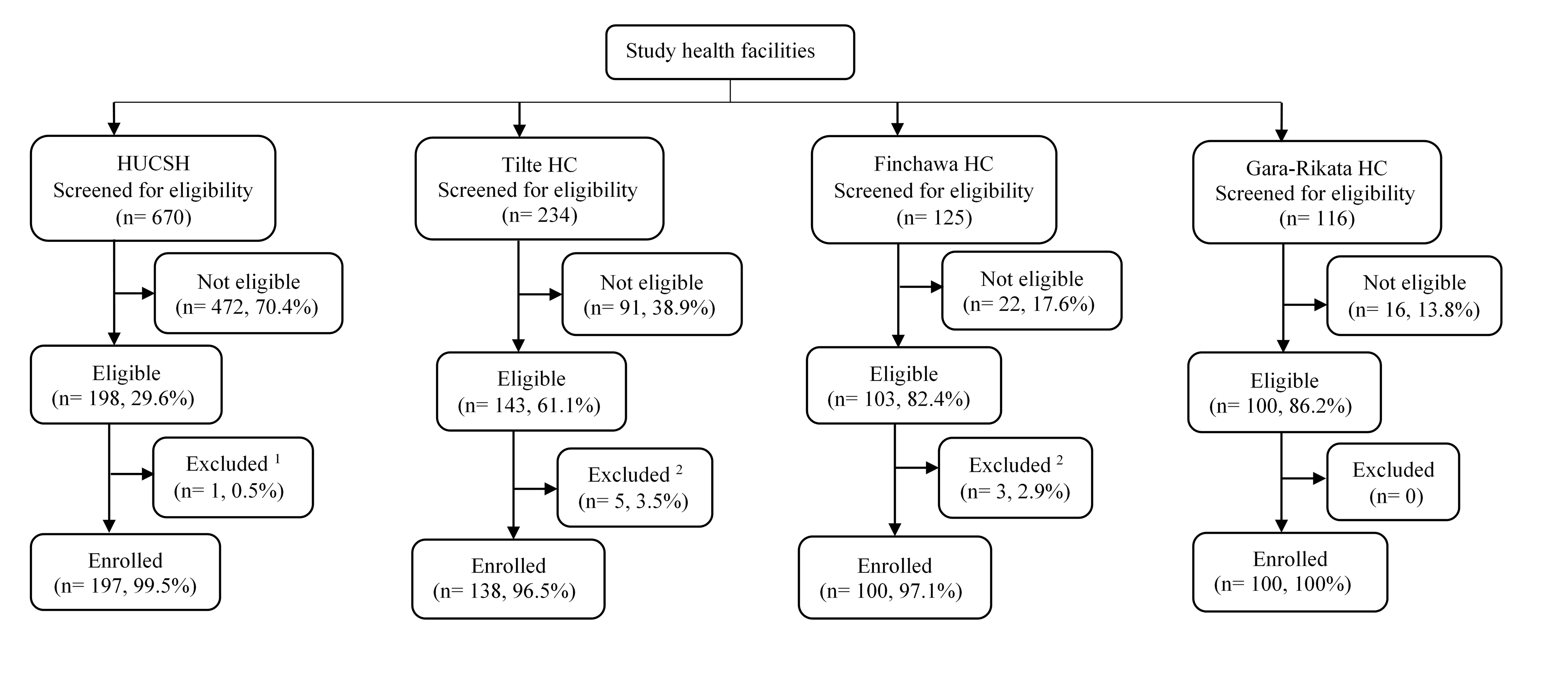

Supplement: S1 Fig — HUCSH, Hawassa University Comprehensive Specialized Hospital; HC; health centre. Reason for exclusion: 1 Skin infection, 2 Urgent referral to higher-level care. (TIF) [file pone.0269725.s001.tif]
