# Supplementary material for: Timely health care seeking and first source of care for acute febrile illness in children in Hawassa, southern Ethiopia
Source: PLoS One. 2022 Jun 9;17(6):e0269725. doi: 10.1371/journal.pone.0269725 (PMC9182269; doi:10.1371/journal.pone.0269725)
Supplement: S1 Table — ⸹Any nearest primary health care facility different from the one first attended 1Travel time to a bypassed primary health care facility by walking. (DOCX) [file pone.0269725.s002.docx]

| **PHC facility** | **First source of care attended** | | | |
| --- | --- | --- | --- | --- |
|  | **Higher-level hospitals**  **n (%)**  **(N= 93)** | **PHC facilities n (%) (N= 400)** | **Private clinic**  **n (%)**  **(N= 42)** | **Total**  **n (%) (N= 535)** |
| **Availability of a nearer PHC than the care first utilized^⸹^** |  |  |  |  |
| Had no nearer PHC | 29 (31.2) | 209 (52.2) | 15 (35.7) | 253 (47.3) |
| Had nearer PHC (bypassed) | 64 (68.8) | 191 (47.8) | 27 (64.3) | 282 (52.7) |
| Health post | 6 (9.4) | 163 (85.3) | 1 (3.7) | 170 (60.3) |
| Health centre | 55 (85.9) | 26 (13.6) | 25 (92.6) | 106 (37.6) |
| Primary hospital | 3 (4.7) | 2 (1.0) | 1 (3.7) | 6 (2.1) |
| **Travel time to a bypassed PHC facility**^1^ **(minute)** | **N= 64** | **N= 191** | **N=27** | **N=282** |
| ≤15 | 35 (54.7) | 158 (82.7) | 14 (51.9) | 207 (73.4) |
| 16 - 30 | 27 (42.2) | 29 (15.2) | 11 (40.7) | 67 (23.8) |
| >30 | 2 (3.1) | 4 (2.1) | 2 (7.4) | 8 (2.8) |
| **Reasons for bypassing** |  |  |  |  |
| Lack of laboratory service | 12 (18.8) | 153 (80.1) | 3 (11.1) | 168 (59.6) |
| Lack of skilled staff | 23 (35.9) | 114 (59.7) | 14 (9.3) | 151 (53.5) |
| Lack of drugs | 9 (14.1) | 117 (61.3) | 6 (22.2) | 132 (46.8) |
| Lack of medical equipment | 19 (29.7) | 63 (33.0) | 7 (25.9) | 89 (31.6) |
| Lack of trust | 20 (31.2) | 47 (24.6) | 12 (44.4) | 79 (28.0) |
| Lack of inpatient service | 9 (14.1) | 5 (2.6) | 3 (11.1) | 17 (6.0) |
| Lack of respect | 2 (3.1) | 8 (4.2) | 4 (14.8) | 14 (5.0) |
