# Supplementary material for: Timely health care seeking and first source of care for acute febrile illness in children in Hawassa, southern Ethiopia
Source: PLoS One. 2022 Jun 9;17(6):e0269725. doi: 10.1371/journal.pone.0269725 (PMC9182269; doi:10.1371/journal.pone.0269725)
Supplement: S2 Table — PHC, primary health care; COR, crude odds ratio; AOR, adjusted odds ratio; CI, confidence interval 1Percentages within categories of the characteristics (raw total). (DOCX) [file pone.0269725.s003.docx]

| Timelines of seeking care | First source of care | Hospitalization | | Total  n (%) | COR (95% CI) | P-value |
| --- | --- | --- | --- | --- | --- | --- |
|  |  | Not hospitalized  n (%)^1^ | Hospitalized  n (%)^1^ |  |  |  |
| Delayed (N=113) | Higher-level hospitals | 22 (42.3) | 30 (57.7) | 52 (46.0) | 1 |  |
|  | PHC facilities | 7 (15.2) | 39 (84.8) | 46 (40.7) | 4.09 (1.54-10.8) | 0.005 |
|  | Private clinics | 2 (13.3) | 13 (86.7) | 15 (13.3) | 4.77 (0.98-23.3) | 0.054 |
| Timely (N=83) | Higher-level hospitals | 14 (35.0) | 26 (65.0) | 40 (48.2) | 1 |  |
|  | PHC facilities | 7 (30.4) | 16 (69.6) | 23 (27.7) | 1.23 (0.41-3.70) | 0.712 |
|  | Private clinics | 6 (30.0) | 14 (70.0) | 20 (24.1) | 1.26 (0.40-3.99) | 0.699 |
