# Supplementary material for: Timely health care seeking and first source of care for acute febrile illness in children in Hawassa, southern Ethiopia
Source: PLoS One. 2022 Jun 9;17(6):e0269725. doi: 10.1371/journal.pone.0269725 (PMC9182269; doi:10.1371/journal.pone.0269725)
Supplement: S1 Appendix — (PDF) [file pone.0269725.s004.pdf]

Place barcode label here



***Form 2: Care-seeking Behaviour Questionnaire*** *(to be completed by Nurse /Health Officer)*

*Participant study number-------------------*

*Name of Health facility-----------------------------------------------*

**Participant contact information** *(This information will be gathered immediately after the caregiver gives consent)*

| | |
|---|---|
| Child's full name | ---------------------------------- Child's card number------------------- |
| Caregiver's full name | ------------------------------------------------ |
| Name of household head | ------------------------------------------------- |
| Caregiver's address: | *Region* :--------------------<br>***1. Rural***<br>*Woreda* (*District* )----------------------- *Kebele* (sub-district) --------------------<br>***2. Urban***<br>*Town* ---------------- *Kebele* (sub-district) --------------------<br>Phone Number (household member):___________________<br>Phone Number (Neighbor): _________________ |

Place barcode label here



**Form 2: Care-seeking Behaviour Questionnaire** *(to be completed by Nurse /Health Officer)*

*Participant study number------------------*

**A. Caregiver's socio-demographic information***(This information will be gathered by interviewing consented caregivers on the day of presentation after the child is managed)*

| | | | |
|---|---|---|---|
| 1. | Date of interview: | __ __(dd)/__ __(mm)/__ __ __ __(yyyy) | |
| 2. | Is the caregiver male or female? | 1. Male | 2. Female |
| 3. | How old is the caregiver? (years) | ☐☐ | |
| 4. | What is the caregiver's relationship to the child? | 1. Biological mother<br>2. Biological father<br>3. Grandmother or grandfather<br>4. Uncle or aunt<br>5. Brother or sister | 6. Other relative<br>7. Not related<br>8. Do not know |
| 5. | What is the caregiver's ethnicity? | 1. Sidama<br>2. Oromo<br>3. Wolayita<br>4. Kembata | 5. Amhara<br>6. Gurage<br>7. Other (Specify)---------- |
| 6. | What is the occupation of the caregiver? | 1. Farmer<br>2. Daily Laborer<br>3. House wife<br>4. Marchant<br>5. Civil servant | 6. Student<br>7. Self-employed<br>8. No work<br>9. Other (Specify)………. |
| 7. | What is the highest level of school attended by the caregiver? | 1. Illiterate<br>2. Read/write but no formal schooling<br>3. First cycle (1-4 grade) | 4. Second cycle (5-8 grade)<br>5. High school (9-12 grade)<br>6. Higher education |
| 8. | What is the religion of the caregiver? | 1. Protestant<br>2. Orthodox<br>3. Muslim | 4. Catholic<br>5. Other (specify)……… |
| 9. | Family size (total number of persons in the household the child belongs) | ☐☐ | |
| 10. | How many under-five children live in the household? | ☐☐ | |
| 11. | Please identify the tools you use to get information? (*Read options for participants)* (*Multiple responses)* Anything else? | 1. Radio<br>2. Television<br>3. News paper<br>4. Non-mobile telephone | 5. Mobile phone<br>6. Mobile internet<br>7. Others (specify)------------ |

Place barcode label here



***Form 2: Care-seeking Behaviour Questionnaire** (to be completed by Nurse /Health Officer)*

*Participant study number------------------*

**B. Caregiver's care-seeking behaviour to the child's illness** *(This information will be gathered by interviewing consented caregivers on the day of presentation after care service is provided)*

Please ask each question and encircle if the participant mentions single or multiple responses among the given options (as instructed under each question). Any participant's response not in the option list would be captured under the option 'other'. Please do not read the options for participants unless specified.

| | | | |
|---|---|---|---|
| 1. | Where do you commonly seek care first for febrile children under-five? (*Single response)* | 1. General hospital<br>2. Primary hospital<br>3. Health center<br>4. Private clinic<br>5. Health post/extension worker | 6.NGO/private hospital<br>7. Private pharmacy<br>8. Religious leader<br>9. Traditional healer<br>10.Other (specify)------- |
| 2. | Why do you think you choose to seek care at that provider? Anything else? (*Multiple responses)* | 1. Availability of qualified staff<br>2. Availability of drugs<br>3. Closest facility<br>4. Availability of laboratory service<br>5. Availability of medical equipment<br>6. Availability of inpatient service<br>7. More familiar with the provider | 8. More trusted service<br>9. Short waiting time<br>10.Affordable cost<br>11.Other (specify)------- |
| 3. | What signs/ symptoms that led you to seek care? Anything else? (*Multiple responses)* | 1. Fever/hot body<br>2. Feeling cold /shivering<br>3. Excessive sweeting<br>4. Vomiting<br>5. Vomiting every thing<br>6. Cough<br>7. Refuse to drink and breastfeed<br>8. Lethargy/unconsciousness | 9. Fast breathing<br>10. Watery diarrhea<br>11. Bloody diarrhea<br>12. Convulsion<br>13. Others (specify)-------- |
| 4. | What do you think the danger signs associated with an urgent need of care? Anything else? (*Multiple responses)* | 1. History of convulsion<br>2. Convulsing now<br>3. Lethargy/unconsciousness<br>4. Unable to drink or breastfeed | 5. Vomiting everything<br>6. Do not know<br>7. Other (specify)---------- |
| 5. | Is the present health provider your first source of care for current child's illness? | 1. Yes → ***Go to Q 11*** | 2. No |
| 6. | If 'No' to Q 5, where was the child seen before? Anything else? (*Multiple responses) (Please indicate order of providers as 1st, 2nd, ….. in case of multiple responses* | 1. General hospital<br>2. Primary hospital<br>3. Health center<br>4. Private clinic<br>5. Health post/extension worker | 6. NGO/private hospital<br>7. Private pharmacy<br>8. Religious leader<br>9. Traditional healer<br>10. Other (specify)------- |
| 7. | Why did you choose that health provider as first source of care? Anything else? (*Multiple responses)* | 1. Availability of qualified staff<br>2. Availability of drugs<br>3. Closest facility<br>4. Quality laboratory service<br>5. Availability of medical equipment<br>6. Availability of inpatient service<br>7. More familiar with the provider | 8. More trusted service<br>9. Short waiting time<br>10. Affordable cost<br>11. Advised by colleague<br>12. Other (specify)------ |

Place barcode label here



***Form 2: Care-seeking Behaviour Questionnaire*** *(to be completed by Nurse /Health Officer)*

*Participant study number-------------------*

| | | | |
|---|---|---|---|
| 8. | Identify the transport means you used to get to that health provider? Anything else? (*Multiple responses)* | 1. Walked<br>2. Motorcycle<br>3. Facility vehicle/ambulance<br>4. Private car<br>5. Bus/minibus | 6. Animal/cart<br>7. Bajaj/taxi<br>8. Other (Specify)------ |
| 9. | How long did it take you to reach that health provider?<br>*Please use the unit 'minute' if less than 1 hour (60 minutes)* | -------------------- Hours or | ------------- Minutes |
| 10. | How much did you spend to seek care at that health provider and went back home? | Transport<br>Lodging/food<br>Medical service<br>***Total*** | ------------- (*Birr*)<br>-------------(*Birr*)<br>-------------(*Birr*)<br>------------(*Birr*) |
| 11. | Why do you choose to seek care at the present health provider? Anything else? (*Multiple responses)* | 1. Availability of qualified staff<br>2. Availability of drugs<br>3. Closest facility<br>4. Availability of laboratory service<br>5. Availability of medical equipment<br>6. Availability of inpatient service<br>7. More familiar with the provider | 8. More trusted service<br>9. Referred from prior care<br>10. Short waiting time<br>11. Affordable cost<br>12. Advised by colleague<br>13. Other (specify)--------- |
| 12. | Identify the transport means you have used to get to the current facility? Anything else? (*Multiple responses)* | 1. Walked<br>2. Motorcycle<br>3. Facility vehicle/ambulance<br>4. Private car | 5. Bus/minibus<br>6. Animal/cart<br>7. Bajaj/taxi<br>8. Other (Specify)------ |
| 13. | How long did it take you to reach the current facility?<br>*Please use the unit 'minute' if less than 1 hour (60 minutes)* | -------------------- Hours or | ------------- Minutes |
| 14. | Is there a closest public primary health care provider to your dwelling than the one you used as first source of care for the current illness? | 1. Yes | 2. No → ***Go to Q 18*** |
| 15. | Which provider is that? *(Read options for participants)* (*Single response)* | 1. Health post/ extension worker<br>2. Health center | 3. Primary hospital |
| 16. | How long does it take you to reach that health provider on walking?<br>*Please use the unit 'minute' if less than 1 hour (60 minutes)* | -------------------- Hours or | ------------- Minutes |

Place barcode label here



***Form 2: Care-seeking Behaviour Questionnaire*** *(to be completed by Nurse /Health Officer)*

*Participant study number-------------------*

| | | | |
|---|---|---|---|
| 17. | Why did not you seek care first from that provider? Anything else? (*Multiple responses)* | 1. Unavailability of qualified staff<br>2. Unavailability of drugs<br>3. Unavailability of laboratory service<br>4. Unavailability of medical equipment<br>5. Unavailability of impatient service<br>6. Lack of general respect | 7. Lack of trust on service<br>8. Long waiting time<br>9. Unaffordable cost<br>10. Other (specify)------ |
| 18. | Have you ever been informed where it is advised to seek health care first if your child get febrile? | 1. Yes | 2. No |
| 19. | How many days back you recognized for the first time the child was febrile? *Please use the unit 'hour' if less than 1 day (24h)* | -------------------- Days or | ------------- Hours |
| 20. | In how many days after onset of fever you sought care at the first formal health provider? *Please use the unit 'hour' if less than 1 day (24h)* | -------------------- Days or | ------------- Hours |
| 21. | *(For caregivers that seek formal health care within 24h of onset of fever)* What are/is your reason/s for care-seeking within the specified time period? *Anything else?* (*Multiple responses)* | 1. Advised by others<br>2. Severe enough<br>3. To avoid complication<br>4. Aware of timely care-seeking | 5. Closer facility<br>6. Past experience<br>7. Other(Specify)------- |
| 22. | *(For caregivers that do not seek formal health care within 24h)* What are/is your reason/s for care-seeking within the specified time period? *Anything else?* (*Multiple responses)* | 1. Unaware of timely care-seeking<br>2. Not severe enough<br>3. No enough money<br>4. Family would not let me<br>5. Busy with other duties | 6. No closer facility<br>7. Poor transportation access<br>8. Other(Specify)------- |

***Name of Interviewer***____________________________ Date Completion: __ __/__ __/__ __ __ __
